# Platelets’ morphology, metabolic profile, exocytosis, and heterotypic aggregation with leukocytes in relation to severity and mortality of COVID-19-patients

**DOI:** 10.3389/fimmu.2022.1022401

**Published:** 2022-11-21

**Authors:** Basma A. Yasseen, Aya A. Elkhodiry, Riem M. El-Messiery, Hajar El-sayed, Malak W. Elbenhawi, Azza G. Kamel, Shaimaa A. Gad, Mona Zidan, Marwa S. Hamza, Mohamed Al-ansary, Engy A. Abdel-Rahman, Sameh S. Ali

**Affiliations:** ^1^ Research Department, Children’s Cancer Hospital Egypt, Cairo, Egypt; ^2^ Infectious Disease Unit, Internal Medicine Department, Faculty of Medicine, Cairo University, Cairo, Egypt; ^3^ Pharmacology Department, Medical Research and Clinical Studies Institute, National Research Center, Cairo, Egypt; ^4^ Department of Clinical Pharmacy Practice, Faculty of Pharmacy, The British University in Egypt, Cairo, Egypt; ^5^ Department of Intensive Care, Faculty of Medicine, Cairo University, Cairo, Egypt; ^6^ Pharmacology Department, Faculty of Medicine, Assuit University, Assuit, Egypt

**Keywords:** Critically ill COVID-19 patients, COVID-19 severity, platelet activation, leukocyte-platelet aggregation, metabolism, mitochondrial function, exocytosis

## Abstract

Roles of platelets during infections surpass the classical thrombus function and are now known to modulate innate immune cells. Leukocyte-platelet aggregations and activation-induced secretome are among factors recently gaining interest but little is known about their interplay with severity and mortality during the course of SARS-Cov-2 infection. The aim of the present work is to follow platelets’ bioenergetics, redox balance, and calcium homeostasis as regulators of leukocyte-platelet interactions in a cohort of COVID-19 patients with variable clinical severity and mortality outcomes. We investigated COVID-19 infection-related changes in platelet counts, activation, morphology (by flow cytometry and electron microscopy), bioenergetics (by Seahorse analyzer), mitochondria function (by high resolution respirometry), intracellular calcium (by flow cytometry), reactive oxygen species (ROS, by flow cytometry), and leukocyte-platelet aggregates (by flow cytometry) in non-intensive care unit (ICU) hospitalized COVID-19 patients (Non-ICU, n=15), ICU-survivors of severe COVID-19 (ICU-S, n=35), non-survivors of severe COVID-19 (ICU-NS, n=60) relative to control subjects (n=31). Additionally, molecular studies were carried out to follow gene and protein expressions of mitochondrial electron transport chain complexes (ETC) in representative samples of isolated platelets from the studied groups. Our results revealed that COVID-19 infection leads to global metabolic depression especially in severe patients despite the lack of significant impacts on levels of mitochondrial ETC genes and proteins. We also report that severe patients’ platelets exhibit hyperpolarized mitochondria and significantly lowered intracellular calcium, concomitantly with increased aggregations with neutrophil. These changes were associated with increased populations of giant platelets and morphological transformations usually correlated with platelets activation and inflammatory signatures, but with impaired exocytosis. Our data suggest that hyperactive platelets with impaired exocytosis may be integral parts in the pathophysiology dictating severity and mortality in COVID-19 patients.

## Introduction

With the recurrent COVID-19 waves entailing hundreds of millions of confirmed cases and accumulated mortality record of > 6.5 million deaths in the summer of 2022, the pandemic is rapidly surpassing the rank of the 7^th^ worst health catastrophe in recorded human history. The increasing consensus that SARS-CoV-2 is here to stay (1) necessitates massive efforts to understand molecular factors contributing to the host immune response leading to severe clinical manifestations and long term maladies. COVID-19-associated coagulopathy is frequently suggested to contribute to thrombosis as well as multi-organ failure leading to severity and mortality (2). In a very recent large-scale clinical study that followed cardiometabolic outcomes up to 12 months after infection (3), cardiovascular events were found to increase early after COVID-19 mainly from pulmonary embolism, atrial arrhythmias, and venous thrombosis.

Numerous reports are now adapting the concept that platelets are integral part of the immune system whether innate or adaptive through a delicate balance between hemostatic and thrombotic functions in response to pathogens (4–6). In fact, evidence showed that platelets’ activation profile during thrombin-regulated hemostatic response to pathogens distinctly differs from that following immune stimulation; e.g. by TLR7-activating virus (7). While hemostatic response involves platelet-platelet interactions, immune-activation leads to smaller platelet groups involving frequent interaction with leukocytes (5). Platelet-leukocyte interactions, especially with neutrophils and monocytes, are thus substantiated as core immune modulators. While some attention has been given to the roles of platelets in COVID-19-associated coagulopathy (8, 9), their modulatory role of the immune response following SARS-CoV-2 infection is not sufficiently studied.

Aggregations of platelets with neutrophils and monocytes are consistently observed in the contexts of a myriad of human conditions and are now considered to constitute one of the most sensitive markers of platelet activation (10). For example, upon activation, platelets bind to neutrophils and trigger the release of cytokines and stimulates the formation of neutrophil extracellular traps (NETs) (11, 12). Ironically, platelet-monocyte aggregation may demark increased inflammation in the pathogenesis of sepsis (13), while suggested to play an anti-inflammatory role in the context of ulcerative colitis (14). The mechanisms promoting the interaction and aggregation of platelets with monocytes or with neutrophils, and the effects of aggregation during the course of SARS-CoV-2 infection are still poorly defined.

Platelets exhibit a diverse metabolic profile with remarkable ability to switch freely between bioenergetic pathways in response to functional demands and dynamic changes in shear stress and coagulations (15, 16). It has been reported that platelets have significant metabolic flexibility, but preferentially rely on glycolysis for ATP synthesis when activated while preserving mitochondrial respiration (15). This was supported by the finding that mitochondrial ATP generation in activated platelets is critical for granule secretion but not essential for aggregation and pro-coagulant phenotypes (17). Nevertheless, platelets’ mitochondria do not only provide bioenergetic supply but are also pivotal to activation and interaction with leukocytes through regulation of calcium and phosphatidylserine externalization (18–20). Studies indicated that the transition of platelets to pro-coagulant phenotype is mediated by hyperpolarized mitochondria (21), extracellular calcium entry (22), and mitochondrial permeability transition pore (mPTP) opening (22) and this is associated with intra-platelet ROS elevation (23). To date, and despite their relevance to the pathology of SARS-CoV-2 infection including clinical outcome, there is a lack of understanding of metabolic reprogramming in platelets of COVID-19 patients or mitochondrial dysfunctions in relation to clinical outcomes in those patients. We designed this study to explore this knowledge gap by addressing the role of mitochondria, ROS, and intracellular calcium in platelets activation and leukocyte interactions in relation to severity and mortality outcomes of COVID-19 patients.

## Methods

### Study design and participants

The present study aims to analyze the effect of COVID-19 severity and mortality on platelet functions in the most severe cases of COVID-19 in comparison with mild/moderate cases and control subjects. This is a prospective observational cohort study of patients with confirmed RT-PCR positive COVID-19. Nasopharyngeal swab RT-PCR results and Lung CT scans were combined to classify severe symptomatic COVID-19 cases. All patients were recruited from Kasr Alainy Cairo University Hospital-ICU-facility at the Internal Medicine Quarantine Hospital. Supportive therapy including supplemental oxygen and symptomatic treatment were administered as required. Hospitalized patients with moderate to severe hypoxia (defined as requiring fraction of inspired oxygen [FiO2] ≥40%) were transferred to the intensive care (ICU) for further management including invasive mechanical ventilation when necessary. ICU-patients recruited in the current study were divided into two arms based on future mortality outcome: Those who survived (ICU-S), and those who died within 20 days of samples collection (ICU-NS).

Patient cohort in this study is an expanded cohort for which we recently reported on the biophysically determined serum albumin damage as an accurate mortality predictor (24). With the initial aim being the assessment of metabolic alterations in platelets, no statistical method was employed to predetermine the number of subjects due to lack of previously reported similar studies on COVID-19 patients. Instead, sample size was based on sample availability and collection continued until a total of N=110 has been reached for COVID-19 patients during the period from October 4, 2020 to December 7 2021. Within the follow-up time 60 patients had died. All available samples were investigated by blinded operators and were all included in the final analysis.

Written informed consents were obtained from participants in accordance with the principles of the Declaration of Helsinki. Children’s Cancer Hospital’s Institutional Review Board (IRB) has evaluated the study design and protocol, IRB number 31-2020 issued on July 6, 2020 and renewed on July 28, 2021.

### Blood samples collection, handling, and processing for platelet analyses

10 mL of fresh peripheral venous blood samples were collected from each subject on ACD tubes. The samples were divided into 2 portions; 2 mL of citrated whole blood that was incubated with RBCs lysis buffer containing NH_4_Cl (ammonium chloride), NaHCO_3_ (sodium bicarbonate), and EDTA (disodium) for 15 min (25) and used for flow cytometry measurements. To be used in metabolic and molecular studies, the remaining 8 mL blood were processed to isolate platelets by differential centrifugation as follows. Platelet-rich plasma (PRP) was obtained by centrifugation at 300× g for 15 min with decreased acceleration and no brakes. PRP was then centrifuged at 4000× g for 10 min. The platelet pellets were resuspended in 1 mL platelet poor plasma and the count was determined using hemocytometer (24, 26). In a few cases, blood samples were disposed because they were clotted either during transfer or during processing.

### Phenotyping of peripheral blood by flow cytometry

Lysed cell suspension was centrifuged at 500xg for 5 minutes, washed twice with phosphate-buffered saline (PBS) and finally, obtained lysed cells were suspended in PBS. Whole blood was assessed for the distribution status of platelets, neutrophils, monocytes and T-T-lymphocytes using 13-color flow cytometer CytoFLEX system (Beckman Coulter Life Sciences CytoFLEX benchtop flow cytometer). Obtained cell suspension was incubated for 30 min in the dark at room temperature with anti-human monoclonal antibodies for identification of cellular subsets: CD-42b-PE (Beckman Coulter Life Sciences, IM1417U) for platelets, CD14-PC7 (Beckman Coulter Life Sciences, A22331) for monocytes, CD66b-APC-Alexa Fluor 750 for neutrophils (Beckman Coulter Life Sciences, B08756), and CD3-ECD (Beckman Coulter Life Sciences, IM2705U) for T-T-lymphocytes. Following incubation, cells were washed with PBS to be suspended in 300 μL and analyzed by flow cytometry for gating platelet-specific CD42b-PE positive population, neutrophil-specific CD66b-APC-Alexa Fluor 750 positive population, monocytes-specific CD14-PE positive population, and T-T-lymphocytes specific CD3-ECD positive population. Data was acquired for 20,000 events and analyzed using CytExpert software to assess the percentage and mean fluorescence intensities (MFIs) of cellular subsets.

### Phenotyping of platelets activation by flow cytometry

Distribution status of activated platelets was measured in whole blood samples by 13-color flow cytometry as described using CytoFLEX system (Beckman Coulter Life Sciences CytoFLEX benchtop flow cytometer). Suspended cells were incubated for 30 min in the dark at room temperature with combinations of anti-human monoclonal antibodies for subset identification as follows: CD-42b-PE (Beckman Coulter Life Sciences, IM1417U) for platelets, PAC1- AF647 (Biolegend, 362806) and CD62P-BV785 (Biolegend, 304942) for activated platelets. Following incubation, cells were washed with PBS to be suspended in 300 μL and analyzed by flow cytometry for gating platelet-specific CD42b-PE positive population and activated platelets PAC1 and CD62P positive population. A number of 20,000 events were acquired and analyzed using CytExpert software to determine the percentage and mean fluorescence intensities (MFIs) of analyzed cell subsets.

### Measurement of ROS by flow cytometry

Whole blood samples were assessed for the generation of ROS by platelets using 2′,7′-dichlorofluorescein-diacetate (DCF, Sigma-Aldrich, D6883). Suspended cells were incubated with DCF (20 μM) and monoclonal antibody; CD-42b-PE (Platelets) for 30 min at room temperature in the dark. Cells were then washed with PBS and resuspended in 300 μL of PBS. A total of 20,000 events were recorded and analyzed using CytExpert program.

### Measurement of transmembrane potential by flow cytometry

The transmembrane potential of different cell populations was measured in whole blood samples using Tetramethylrhodamine methyl ester perchlorate (TMRM, Sigma, T5428). Suspended cells were incubated with TMRM (1 μM) and combinations of monoclonal antibody; CD-42b-BV650 (Platelets) for 30 min at room temperature in the dark. Cells were then washed with PBS and resuspended in 300 μL of PBS. A total of 20,000 events were recorded and analyzed using CytExpert program.

### Measurement of intracellular calcium by flow cytometry

Intracellular calcium of different cell populations was measured in whole blood samples using Fluo-4-AM, cell permeant (Fluo-4-AM, ThermoFisher scientific, F14201). Suspended cells were incubated with Fluo-4-AM (10 μM) in the presence of pluronic F-127 (0.02%) for 15 min followed by addition of combinations of monoclonal antibody; CD-42b-PE (Platelets) in HEPES buffer for another 15 min at room temperature in the dark. Cells were then washed with HEPES buffer and resuspended in 300 μL of HEPES buffer and incubated for 20 min. A total of 20,000 events were recorded and analyzed using CytExpert program.

### Measurements of mitochondrial respiratory rates

Assessment of mitochondrial respiratory functions was carried out at 37 °C using high-resolution respirometry system Oxygraph-2K (Oroboros Instruments, Innsbruck, Austria) as previously described (26)). Prior to starting the experiment, oxygen calibration was done by permitting the respiration medium MIR05 to equilibrate with air in the oxygraph chambers till the detection of a stable signal. Platelets were then transferred to chambers and permeabilization was performed by addition of 50 µg/mL saponin. Substrate–uncoupler–inhibitor titration (SUIT) protocol was applied as follows: pyruvate (5 mM), malate (5 mM), (PM) adenosine diphosphate (ADP, 1 mM), glutamate (G, 5 mM), succinate (S, 10 mM), oligomycin (1 μg/mL) carbonyl cyanide-4-(trifluoromethoxy) phenylhydrazone (FCCP) (multiple 0.5 µM infusions), rotenone (R, 2 µM), Antimycin A (AmA, 1.25 µM), N,N,N’,N’-tetramethyl-p-phenylenediamine (0.5 mM)/Ascorbate (2 mM), (TMPD/Asc). The rates of oxygen consumptions were normalized to platelets count and estimated as the negative time derivative of oxygen concentration. Mitochondrial respiratory parameters were assessed as follow (1): Oxidative phosphorylation (OXPHOS) rate after adding saturating ADP in the presence of pyruvate, malate and/or glutamate (OXPHOS-I) or in the presence of succinate as a complex II substrate (OXPHOS I + II) (2). Electron transfer system (ETS) capacity maximum respiration rate: in the presence of FCCP (3). Complex IV activity after addition of TMPD/ASC. DatLabVR software version 7.4.0.4 (Oroboros Instruments, Innsbruck, Austria) was utilized for data acquisition and analysis.

### Citrate synthase activity

Samples from the Oroboros O2k chambers were collected at the end of each experiment and stored in -80°C until measurement. Citrate synthase activity was assessed as previously described (27) using 300 μg of platelets protein. Since the irreversible chemical reaction CoA-SH + DTNB → TNB + CoA-S-S-TNB is catalyzed by citrate synthase, the amount of thionitrobenzoic acid (TNB) was quantified by measuring absorbance at 412 nm using Cytation5 Cell Imaging Reader (Agilent BioTek, CA, USA).

### Measurement of Oxygen Consumption Rate (OCR) and Extracellular Acidification Rate (ECAR) in platelets

Platelets bioenergetics were measured through oxygen consumption rate (OCR) and proton production using XF analysis (XF96, Seahorse analyzer, Agilent) as previously described (28–30). In brief, For the mitochondrial stress test: 2 × 10^7^ platelets/well were plated on 96 well format XF plates in unbuffered Dulbecco’s Modified Eagle’s Media (DMEM; with 1 mM pyruvate, 5.5 mM D-glucose, 4 mM L-glutamine, pH 7.4 at 37°C). For the glycolysis stress test, the same seeding density was applied but in unbuffered Dulbecco’s Modified Eagle’s Media (DMEM; with 4 mM L-glutamine, pH 7.4 at 37°C). Plates was subsequently centrifuged (800g for 5 minutes) to sediment platelets’ monolayers in wells. Plates were allowed to settle and were incubated for 30-40 min at 37 °C in non- CO_2_ incubator. OCR and the extracellular acidification rate (ECAR) were measured simultaneously for 30 min to establish a baseline measurement. The mitochondrial stress test was performed by measuring OCR during sequential injection of oligomycin (2.5 µM), carbonyl cyanide p-(trifluoro-methoxy) phenyl-hydrazone (FCCP) (2 µM) and rotenone: antimycin A mixture (2.5 µM). For the glycolysis stress test, ECAR measurements were analyzed during the following injections sequence: glucose (5.5 mM), oligomycin (2.5 µM), 2-deoxy-D-glucose (2-DG) (50mm). All measurements were normalized to seeding cell density. Proton leak was quantified as the rate of respiration in the presence of rotenone and antimycin A subtracted from the rate of respiration in the presence of oligomycin. ATP-linked respiration was the difference of rate in basal respiration and oligomycin. Non-mitochondrial oxygen consumption is the rate of respiration in the presence of rotenone. Glycolysis, glycolytic capacity, and glycolytic reserve were calculated by subtracting the average rates before and after the addition of glucose, ATP synthase inhibitor oligomycin and 2-DG.

### Electron microscopy

10 mL fresh peripheral citrated blood samples were centrifuged at 800g for 5 minutes without brakes. The plasma top layer was removed leaving less than 1 mL on top of the buffy coat. Cold fixative containing 82 mM sodium monophosphate, 20 mM sodium hydroxide, 4% formaldehyde and 0.01% glutaraldehyde, pH 7.2, was added drop wise on top of the sample and left for 30 minutes. The coat was then transferred into the same fixative for short storage at 4°C. Fixed samples were processed for the transmission electron microscopy (TEM) as described previously (31). The ultrathin sections were post-stained in saturated uranyl acetate and lead citrate, and examined by JSM1400 plus-JEOL transmission electron microscope (Alexandria university microscopy unit). Fixed samples that were stored for scanning electron microscopy (SEM) were processed and coated using Sputter Coating Evaporator as described in (32, 33). JSM-5300 – JEOL scanning electron microscope (Alexandria university microscopy unit) was used to obtain SEM images.

### Western blot analyses

Platelets were incubated with protein lysis buffer containing a proteinase and phosphatase inhibitor cocktail for 15 minutes to prepare protein lysates. 60 µg of lysed proteins were loaded, resolved by 15% SDSPAGE and transferred to PVDF membranes. Membranes were blocked with 5% nonfat dry milk for 1 h and then incubated with mouse Total OXPHOS Human WB Antibody Cocktail (1: 500) (Abcam, ab110411) at 4°C overnight. Membranes were then probed with anti-mouse HRP conjugated secondary antibody for 1 h (1:5000) and developed using the enhanced chemiluminescence (ECL) reagent (Pierce). Membranes were stripped for the detection of β-actin protein using anti- b-actin (1:1000). Bands were quantified by densitometric analyses using Image Lab Software version 6.1.0 (Bio-rad Laboratories, California, USA). The density of each band was normalized to β-actin band density.

### Quantitative Polymerase Chain Reaction (qPCR)

qPCR was performed to assess complex I and complex IV gene expression in platelet samples isolated from healthy controls and COVID-19 infected patients. RNA was isolated using Trizol reagent (ThermoFisher, 15596026) followed by RNA purification using miRNeasy Mini Kit (Qiagen, 217004). RNA concentration and purity was measured using nanodrop and cDNA reverse transcription was carried out for total RNA using Quantitect Reverse Transcription kit (Qiagen, 205311). qPCR was carried out using primer-specific annealing temperature on cDNA templates. Primers spanning exon-exon junctions specific for complex I (NDUFA4), complex IV (MTCO2) and β-actin were used as listed in **Table 1**. 2x Maxima™ SYBR™ Green Master Mix (Applied Biosystems, k0251) was used to perform qPCR on QuantStudio Real-Time PCR (QuantStudio 12K Flex Real-Time PCR System). Levels of RNA were normalized to β-actin levels and estimated as delta-delta threshold cycle (ΔΔCT). Each sample was measured 3 times and average was calculated.

**Table 1:** Primer sequences used for qPCR reactions.

NDUFA4 Primers	Forward	5’ AAGCATCCGAGCTTGATCCC 3’
	Reverse	5’ ACAATGCCAGACGCAAGAGA 3’
MTCO2 primers	Forward	5’ CGTCTGAACTATCCTGCCCG 3’
	Reverse	5’ GGGATCGTTGACCTCGTCTG 3’
β-actin primers	Forward	5’ CACCATTGGCAATGAGCGGTTC 3’
	Reverse	5’ AGGTCTTTGCGGATGTCCACGT 3’

### Statistical analysis

Statistical analysis and data graphing were performed using OriginPro 2021 (OriginLab Corporation, Northampton, USA). Graphical representations of data utilized distribution and rug analysis which visualize prevalence of a given parameter among studied subjects (subject counts) while showing the actual scatter of data points over vertical-dash rugs on the *x* axis. Violin plots were also utilized to outline statistical analyses outcomes and to demonstrate data scatter, distribution, and mean ± SD or SEM as stated in figure legends. Exact p-values are given for each comparison on most graphs and in text. Continuous variables were expressed as mean and standard deviation or as median (IQR). Following tests for normality (Shapiro–Wilk test), variables that fulfilled the normality test were analyzed using the ANOVA test while non-normally distributed data was analyzed using the Kruskal–Wallis test. Both ANOVA and Kruskal–Wallis test were followed by Tukey *post-hoc* tests to compare the differences between the three groups. Categorical variables are reported as counts and percentages while continuous variables are expressed as mean ± SD. Differences between percentages were assessed by Pearson’s χ^2^ tests or Fisher exact tests when the number of observations per group were less than 5. The χ^2^ tests provided results that tested the hypothesis that the mortality and a given variable (e.g. sex or a comorbidity) are independent. When *p* is less than the significant level of 0.05, there is significant evidence of association between mortality and the variable.

### Data sharing statement

Original datasets and detailed protocols are available upon request from the corresponding author sameh.ali@57357.org.

## Results

### Demographic, clinical, and laboratory hematologic characteristics of the studied COVID-19 patients

The demographic and clinical characteristics of the studied patients are shown in **Table 2**. In the present study, participants were divided into three groups; Non- ICU patients (n=15), ICU- Survivors (ICU-S, n=35) and ICU-Non survivors (ICU-NS, n=60). No clinical or demographic characteristic showed statistically significant difference between the three groups except for the age (p =0.004), and blood saturation level (p=0.003). The number of patients who are suffering from diabetes and/or cardiovascular diseases are significantly higher in the ICU-NS group (p <0.05) than the other groups. Moreover, the number of patients who were treated with steroids, remdesivir, hydroxychloroquine and/or carbapenem antibiotics was significantly higher in the ICU-NS group than in the other 2 groups (p <0.05).

**Table 2:** Demographic and clinical characteristics of the participants.

	NON-ICUn (%)	ICU-Sn (%)	ICU-NSn (%)	p value
Male	6 (40%)	22 (62.8%)	34 (56.6%)	0.33
Age	50 (36–59)	61 (55–68)	67.5 (53.5-75.25)	**0.004‡**
sO2	95 (93.5-6.5)	94 (83.5-97)	88 (71–92)	**0.003‡**
Hypertension	2 (13.3%)	10 (31.25%)	27 (45%)	0.057
Diabetes	3 (20%)	6 (17.14%)	26 (43.3%)	**0.017**
Cardiovascular diseases	0	3 (8.57%)	15 (25%)	**0.02**
Cancer	0	4 (11.4%)	5 (8.3%)	0.40
Asthma	1 (6.6%)	2 (5.7%)	4 (6.6%)	0.98
Insulin	2 (13.3%)	6 (17.14%)	11 (18.33%)	0.90
Anticoagulant	10 (66.66%)	16 (45.71%)	29 (48.33%)	0.37
Steroids	9 (60%)	11 (31.42%)	34 (56.66%)	**0.04**
Hydroxychloroquine	6 (40%)	1 (2.85%)	2 (3.33%)	**< 0.001**
IL6 inhibitors	0	3 (8.57)	7 (11.66%)	0.37
Remdesivir	1 (6.66%)	3 (8.57%)	20 (18.18%)	**0.04**
Ivermectin	2 (13.3%)	1 (2.85%)	6 (10%)	0.34
carbapenem antibiotics	3 (20%)	11 (31.42%)	31 (51.66%)	**0.03**
Fluoroquinolone	2 (13.33%)	7 (20%)	18 (30%)	0.31
Oxazolidinone antibiotic	1 (6.66%)	7 (20%)	13 (21.66%)	0.41

sO2, blood oxygen saturation level; IL-6, interleukin-6. Data are expressed as frequency (%). Comparisons were conducted using the Pearson’s χ2 test; ‡ p value was obtained through Kruskal–Walli’s test. Values in bold represent statistically significant comparisons, p < 0.05.

**Table 3** shows laboratory results obtained for participants at the site of sample collection during hospitalization. Although when comparing all parameters in the three groups, we observed changes following similar reported results in previous studies, mean comparisons by Tukey test reported non-significant changes in all parameters except for a significant decrease in platelet counts (p <0.001), and albumin levels (p <0.001). Moreover, blood analyses showed significant increases in the white blood cell counts (p <0.001), neutrophil counts (p=0.015), C-reactive protein levels (p=0.02), ferritin levels (p=0.004),D-Dimer levels (p=0.001), and Creatinine levels (p=0.01) in critically-ill patients relative to mild/moderate patients.

**Table 3:** Laboratory results of participants.

	NON-ICU	ICU-S	ICU-NS	P-value
WBCs (×103/ml)	7.05 (4.48-11.72)	9.5 (7.73-12.5)	13.19 (9.12-17.36)	**< 0.001**
Platelets (106/ml)	324.07+98.54	274.5+115.02	208.65+112.77	**0.001‡**
T-lymphocytes	1.44 (0.29-3.24)	3.45 (1.05-8.92)	2.6 (0.94-5.1)	0.25
Monocytes	0.65 (0.22-2.5)	0.98 (0.42-5.02)	1.43 (0.81-5.67)	0.33
Neutrophils	2.49 (1.56-18.84)	32.72 (19.6-78.45)	18.56 (9.2-35.2)	**0.015**
INR	1 (1-1.3)	1.12 (1.01-1.37)	1.2 (1.07-1.355)	0.18
CRP (mg/L)	23.88 (13-111.5)	56.5 (13.45-126.42)	108.06 (45–175)	**0.02**
D-Dimer (mg/ml)	0.51 (0.19-1.53)	0.98 (0.2-2.95)	2.2 (1.1-4.8)	**0.001**
IL-6 (pg/ml)	324 (35–613)	25 (4.24-885)	77.55 (20.92-244)	0.66
Ferritin	285 (36.5-1306.1)	797 (476.5-954)	1149 (696.5-2000)	**0.004**
Albumin	3.65 (2.85-3.9)	2.75 (2.5-3.25)	2.5 (2.15-2.9)	**< 0.001**
haemoglobin (g/dl)	12.8 (10.55-14.25)	11.7 (9.12-13.42)	11.1 (9.6-12.4)	0.16
ALT (U/L)	30 (12.5-60.5)	25 (16–64)	24 (17.5-40.5)	0.97
AST (U/L)	19 (16.5-42)	30.5 (20.5-45.25)	35 (26–50)	0.07
Creatinine (mg/dL)	0.85 (0.6-0.975)	0.96 (0.75-1.23)	1.28 (0.8-1.42)	**0.01**

ALT, alanine transaminase; AST, aspartate transaminase; CRP, high-sensitivity C-reactive protein; IL-6, interleukin-6; INR, international normalized ratio; WBC, white blood cell. Data are expressed as median (IQR). Comparisons were conducted using the Kruskal–Walli’s test for continuous variables and; ‡ p value was obtained through ANOVA test. Values in bold represent statistically significant comparisons, p < 0.05..

### Morphological alterations in platelets of critically ill patients demonstrated by flowcytometry and transmission electron microscopy

**Figure 1:**
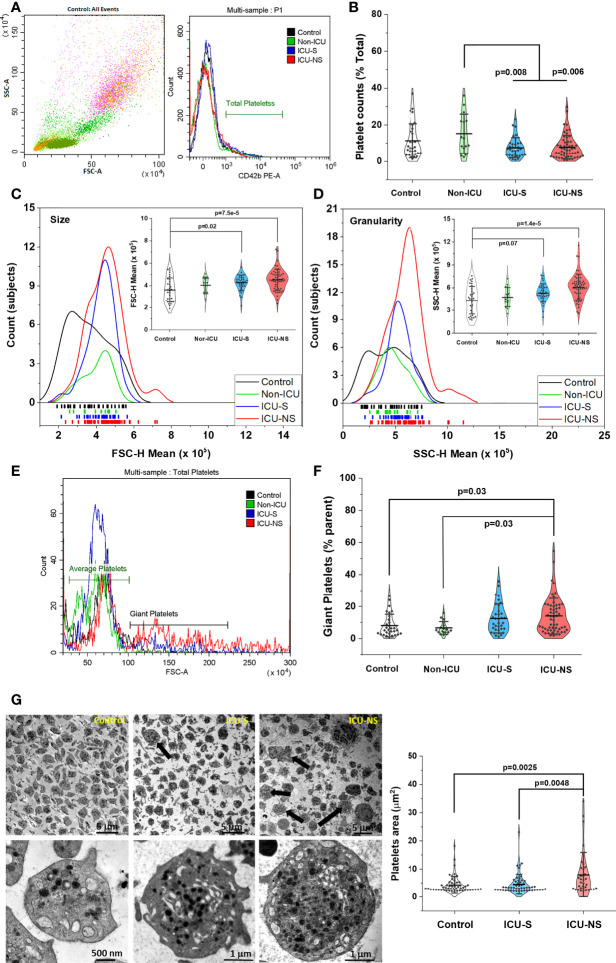
Increased size, granularity, and giant platelets counts are hallmarks in severe COVID-19 patients. Identification of platelets, determination of their counts, size and granularity by whole blood flow cytometry. A discriminator is set to only record events that express a platelet-specific marker (i.e., CD42b). Events are double gated for characteristic light scatter and platelet-specific marker; CD42b expression **(A)**. **(B)** A scatter and violin diagram showing statistically significant decrease in platelet counts of ICU-hospitalized (survivors, and non-survivors) when compared to non-ICU (mild+moderate) patients (n=31, 15, 34, and 56 for control, non-ICU, ICU-S, ICU-NS; respectively). **(C)** A distribution and rug plot showing a systematic increase in the mean platelet size with COVID-19 severity. A violin plot within **(C)** showing a statistically significant increase in the mean FSC (platelet size) in ICU-hospitalized patients relative to control group (n=31, 15, 35, and 57 for control, non-ICU, ICU-S, ICU-NS; respectively). **(D)** A diagram revealing increased granularity in platelets of non-survivors relative to control platelets (n=31, 15, 35, and 57 for control, non-ICU, ICU-S, ICU-NS; respectively). **(E)** Representative flow cytometric histograms comparing populations of giant platelets for all groups. **(F)** When the percentage of giant platelets were compared for all groups, non-survivors’ platelets comprises significantly larger subset of giant platelets (n=30, 15, 35, and 56 for control, non-ICU, ICU-S, ICU-NS; respectively). **(G)** Representative TEM images for all groups, showing ultrastructural features of peripheral blood cells in fields populated with platelets. The upper-right image and quantification panel revealed more frequently observed giant platelets in the ICU-NS group (n=3 of each group and analyzed platelets = 54, 53, 34 for control, ICU-S, and ICU-NS; respectively). Also, platelets exhibited morphological changes from a resting discoid shape in control subjects to an activated state with numerous pseudopodia in severe COVID-19 patients. Additional images are given in **Supplementary Figure S3**. Multiple comparisons were carried out using ANOVA followed by Tukey test and p values are given. Data plotted as mean ± SD.

COVID-19 pathology involves the impairment of the circulatory system including vascular occlusion, hypercoagulability, and reduced oxygen capacity. Platelet count, volume, and morphology are established risk factors for cardiovascular events. We explored if these parameters exhibit systematic changes in COVID-19 patients with variable severity and mortality outcomes. Platelet count was significantly lower in critically-ill patients when compared with mild/moderate (Non-ICU) group (Non-ICU vs. ICU-S, p=0.008; Non-ICU vs. ICU-NS, p=0.006) but those patients exhibited a weak trend of decreasing platelet populations relative to control group as determined by the proportion of CD42b-positive cells (% Total); **Figures 1A, B** (Mean ± SEM, control, n=31: 11.37 ± 1.68, non-ICU, n= 15: 15.22 ± 2.76, ICU-S, n=34: 7.63 ± 0.94, ICU-NS, n=56: 7.89 ± 0.87). This result is similar to those obtained through absolute platelet counts carried out at the site of sample collection (**Table 3**). We then compared the mean platelet volume as estimated by the forward scatter analysis using flow cytometry (34). **Figure 1C** shows a distribution and rug plot comparing subject counts and FSC mean value distributions. In the lower part of the graph, the rugs reveal individual subjects’ distribution in terms of their platelet FSC-H parameter. It is clearly noticeable that the mean platelet size increases systematically with COVID-19 severity (Mean ± SEM, control, n=31: 3.60x10^5^ ± 0.20x10^5^, non-ICU, n=15: 4.02x10^5^ ± 0.19x10^5^, ICU-S, n=35: 4.30x10^5^ ± 0.12x10^5^, ICU-NS, n=57: 4.51x10^5^ ± 0.12x10^5^). The inset within **Figure 1C** is a violin plot showing the probability density of the FSC data for different groups as smoothed by a kernel density estimator. The violin plot includes all analyzed data with a marker for the mean FSC along with whiskers indicating the standard deviations. Our data analysis revealed a statistically significant increase in the mean FSC (platelet size) in ICU-hospitalized patients relative to control group (ICU-S vs. control, p=0.02; ICU-NS vs. control, p=7.5x10^-5^).

We then used side scatter (SSC-H) measurement to provide information about, and compare the internal complexity (i.e. granularity) of platelets in all groups, **Figure 1D**. Platelets of non-survivors exhibit particularly increased granularity relative to control platelets (ICU-NS vs. control, p=1.4 x10^-5^; Mean ± SEM, control, n=31: 4.40x10^5^ ± 0.33x10^5^, non-ICU, n=15: 4.77x10^5^ ± 0.33x10^5^, ICU-S, n=35: 5.32x10^5^ ± 0.20x10^5^, ICU-NS, n=57: 6.09x10^5^ ± 0.22x10^5^). These results enticed us to further analyze our flow cytometry data looking for populations of giant platelets. The presence of large platelets is usually associated with enhanced platelet turnover which is a hallmark of inflammatory response and correlates with an increased ischemic risk (35, 36). We compared the percentage of platelets exhibiting 2-5 times larger mean sizes as reflected in their FSC values (**Figure 1E**) and found that non-survivors’ platelets include significantly greater subset of giant platelets in **Figure 1F** (ICU-NS vs. control, p=0.03; Mean ± SEM, control, n=30: 8.13 ± 1.29, non-ICU, n= 15: 6.59 ± 1.03, ICU-S, n=35: 12.51 ± 1.54, ICU-NS, n=56: 14.02 ± 1.50).

To confirm the presence of these morphological changes we used transmission electron microscopy (TEM) to assess ultrastructural features of peripheral blood cells (buffy coats) from control and ICU-hospitalized survivors and non-survivors COVID-19 patients. Fields populated with platelets were imaged and representative images are given and analyzed in **Figure 1G** (Mean ± SEM, control, n of platelets analyzed=54: 4.39 ± 0.41, ICU-S, n=53: 4.59 ± 0.49, ICU-NS, n=34: 7.98 ± 1.35). As can be seen in this figure, giant platelets are more frequently encountered in the ICU-NS group (upper-right image and quantification panel, ICU-NS vs. control, p=0.0048, where n=3 subjects from each group). Moreover, platelets generally show a shift from a resting discoid shape in control subjects to an activated state with numerous pseudopodia in severe COVID-19 patients. In tune with the flow cytometry results in **Figure 1D**, enlarged TEM images in the lower row of **Figure 1G** indicate a remarkably increased granularity; i.e. platelet internal complexity especially in non-survivors relative to both control and survivor groups.

### COVID-19 severity and mortality are associated with metabolic depression and diminished bioenergetics flexibility in isolated platelets

**Figure 2:**
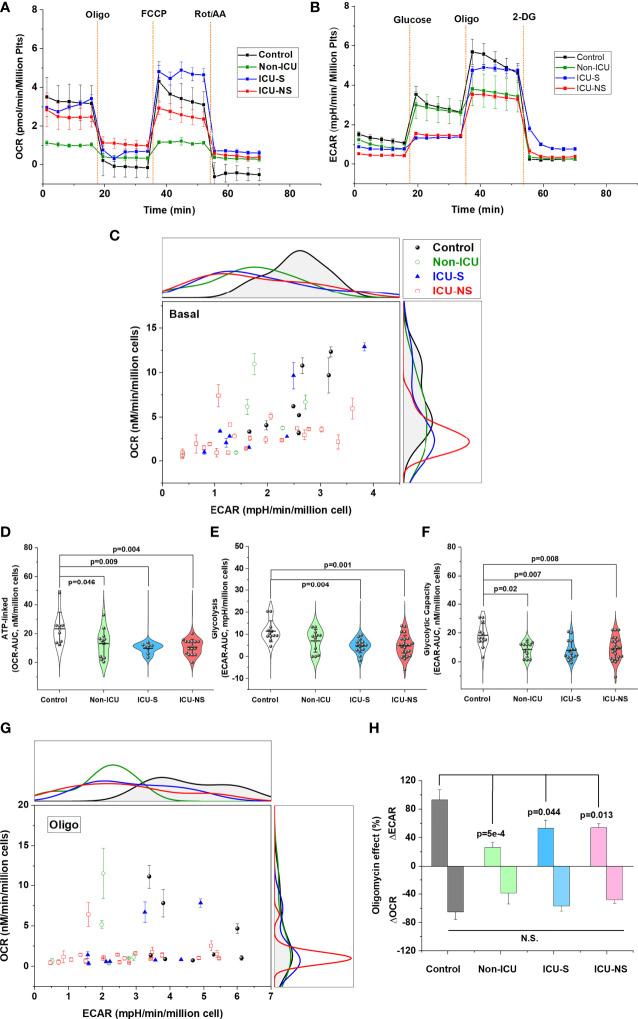
Metabolic profiling of isolated platelets showed remarkable metabolic depression and impaired metabolic flexibility in severe COVID-19 patients. The OCR (aerobic metabolism) **(A)** and ECAR (glycolytic flux) **(B)** were measured in platelets freshly isolated from control, non-ICU, ICU-S, and ICU-NS and normalized to platelets count. **(C)** ECAR and OCR data were plotted simultaneously to reveal overall relative basal metabolic profiles (no substrate added) for all groups. Metabolic analysis of basal activities showing diminished glycolytic as well as mitochondrial respiratory activities in platelets isolated from ICU-hospitalized COVID-19 patients (n=8, 6, 8, and 21 for control, non-ICU, ICU-S, ICU-NS; respectively). **(D)** When ATP-linked oxygen consumption were compared for all groups, both non-ICU and ICU groups showed statistically significant metabolic depression relative to control groups (n=8, 11, 8, and 15 for control, non-ICU, ICU-S, ICU-NS; respectively). Glycolytic metabolism **(E)** and glycolytic capacity **(F)** were impaired in patients’ platelets compared to control platelets (n=12, 11, 15, and 23 for control, non-ICU, ICU-S, ICU-NS; respectively). **(G)** ECAR and OCR data were plotted concurrently to compare metabolic flexibility under the inhibition of mitochondrial ATP-synthesis by oligomycin for all groups (n=8, 6, 8, and 21 for control, non-ICU, ICU-S, ICU-NS; respectively). **(H)** A diagram showing significantly impaired metabolic switch to glycolytic pathway in COVID-19 patients compared to control (ΔECAR, n=7, 6, 7, and 19 for control, non-ICU, ICU-S, ICU-NS; respectively; ΔOCR, n=7, 6, 8, and 21 for control, non-ICU, ICU-S, ICU-NS; respectively). Multiple comparisons were carried out using ANOVA followed by Tukey test and p values are given.

Metabolic characteristics dictate platelet function and activation (15, 16), but this has not been unambiguously demonstrated in the context of COVID-19 pathology. We asked whether severity and mortality of COVID-19 patients are associated with altered glucose metabolism including oxidative phosphorylation and glycolysis. Seahorse metabolic analysis of freshly isolated platelets from representative subsets from all groups revealed an overall COVID-19-associated depression in aerobic and anaerobic pathways, **Figures 2A–C**. That is, despite large variability of basal metabolic activities; i.e., without adding external substrates ICU-hospitalized COVID-19 patients exhibit lower glycolytic as well as mitochondrial respiratory activities (**Figure 2C**). This metabolic depression reached statistical significance during ATP-linked oxygen consumption even in non-ICU patients indicating defective mitochondrial bioenergetics at early onset of SARS-CoV-2 infection (**Figure 2D**, Non-ICU vs. control, p=0.046; ICU-S vs. control, p=0.009; ICU-NS vs. control, p=0.004, where Mean ± SEM, control, n=8: 23.31 ± 4.08, non-ICU, n=11: 13.16 ± 3.05, ICU-S, n=8: 9.85 ± 1.23, ICU-NS, n=15: 10.56 ± 1.39). Relative to control platelets, glycolytic metabolism and glycolytic capacity were also lower in patients’ platelets (**Figure 2E**, ICU-S vs. control, p=0.004; ICU-NS vs. control, p=0.001, where Mean ± SEM, control, n=12: 11.37 ± 1.37, non-ICU, n=11: 5.17 ± 1.56, ICU-S, n=15: 3.09 ± 0.8, ICU-NS, n=23: 4.92 ± 1.03) (**Figure 2F**, Non-ICU vs. control, p=0.02; ICU-S vs. control, p=0.007; ICU-NS vs. control, p=0.008, where Mean ± SEM, control, n=12: 18.22 ± 2.41, non-ICU, n=11: 8.42 ± 1.58, ICU-S, n=15: 7.85 ± 1.74, ICU-NS, n=23: 8.88 ± 1.72).

A relevant aspect of platelets’ metabolic phenotype is their metabolic flexibility; i.e. the capacity to switch among energy substrates to generate ATP in response to changes in functional bioenergetic demands. For instance, transition of platelets to an activated state was described to involve a shift to a glycolytic phenotype (15). We analyzed the association of COVID-19 infection severity and mortality outcomes in terms of the ability of glucose-supplemented platelets to switch to glycolytic pathway under the inhibition of mitochondrial ATP-synthesis by oligomycin, **Figures 2G, H**. While platelets of normal control subjects were able to remarkably increase their ECAR with > 90% in response to around 60% oligomycin-induced mitochondrial inhibition, those from COVID-19 patients showed significantly diminished ability to compensate for the induced bioenergetic shortage following ATP synthase inhibition, **Figure 2H** (Non-ICU vs. control, p=5x10^-4^; ICU-S vs. control, p=0.044; ICU-NS vs. control, p=0.013, where changes in Mean ± SEM (% of ECAR without oligomycin), control, n=7: 93.03 ± 14.4, non-ICU, n=6: 26.36 ± 6.9, ICU-S, n=7: 53.14 ± 11.36, ICU-NS, n=19: 53.98 ± 5.47).

### Critically-ill patients’ platelets exhibit impaired mitochondrial respiratory enzyme activities and swelled mitochondria

**Figure 3:**
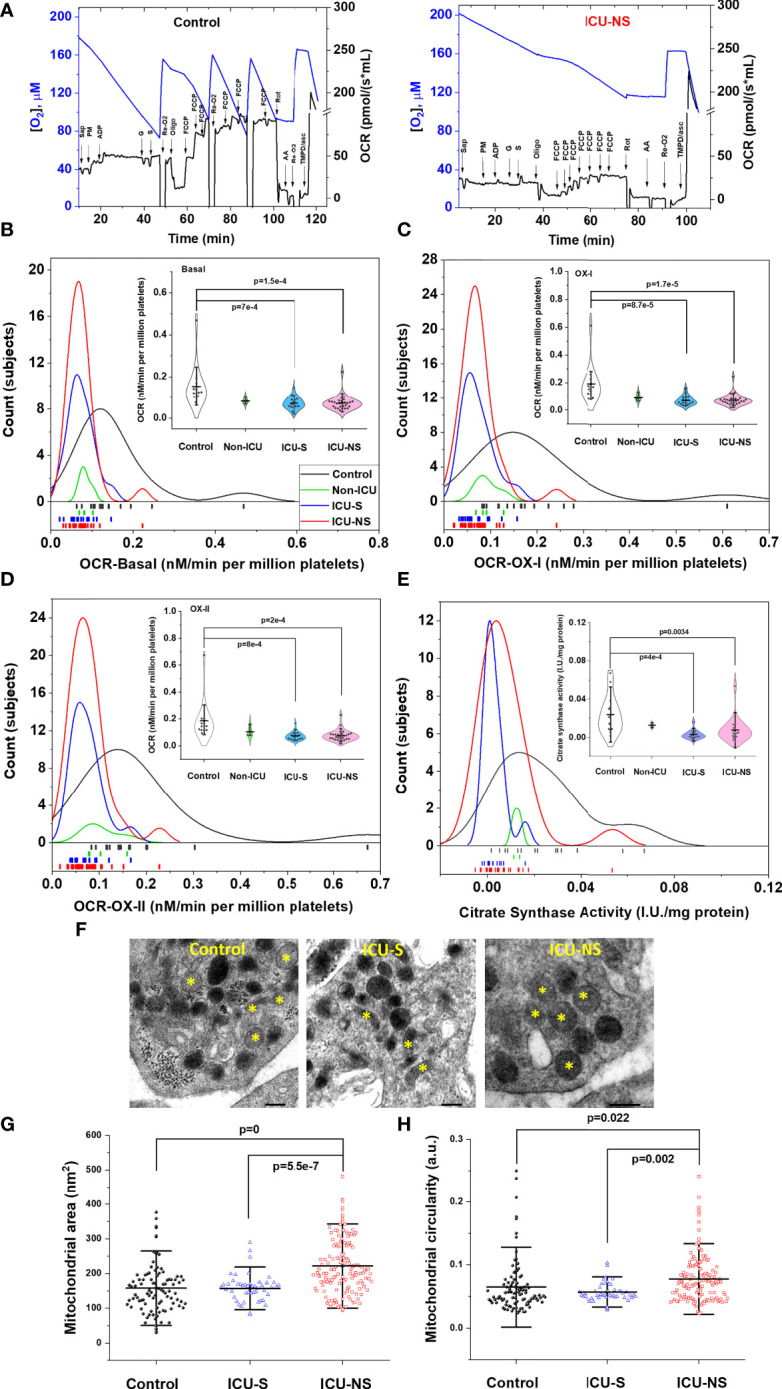
Depressed mitochondrial respiratory activity in severe COVID-19 patients. **(A)** Representative figures of the O_2_ flux per volume measured by high-resolution OROBOROS O2k respirometer in permeabilized platelets for a control and an ICU-NS subjects. Distribution and rug plots showing reduction in mitochondrial respiration of ICU-hospitalized patients’ platelets at basal **(B)** or upon Complex-I **(C)** or complex-II **(D)** stimulations. Violin plots within **(B–D)** showing a statistically significant decrease in the oxygen consumption rate at basal **(B)** or during Complex-I **(C)** or complex-II stimulations **(D)** in ICU-hospitalized patients relative to control group (n=15, 4, 17, and 29 for control, non-ICU, ICU-S, ICU-NS; respectively). **(E)** Citrate synthase activity in platelets was significantly decreased in ICU-hospitalized patients relative to control group (n=15, 2, 15, and 21 for control, non-ICU, ICU-S, ICU-NS; respectively). **(F)** Representative TEM images showing changes in mitochondria morphology in platelets of control and ICU-hospitalized patients. Additional images are given in **Supplementary Figure S3**. Quantification diagrams revealed significantly increased average cross-sectional area **(G)** and circularity **(H)** in platelets mitochondria from the ICU-NS group relative to control and ICU-S. groups (n=1-3 for each group and analyzed mitochondria n= 96, 48, 144 for control, ICU-S, ICU-NS; respectively). Multiple comparisons were carried out using ANOVA followed by Tukey test and p values are given. Data plotted as mean ± SD.* denotes identified mitochondria.

Since mitochondrial ATP synthesis in activated platelets is consistently reported to regulate granule secretion [e.g (17).], we dissected mitochondrial respiratory function using high-resolution respirometry (**Figure 3**). In tune with the Seahorse data (**Figure 2**), mitochondrial respiration of ICU-hospitalized patients’ platelets was remarkably reduced whether at basal (**Figure 3B**, ICU-S vs. control, p=7x10^-4^; ICU-NS vs. control, p=1.5x10^-4^, where Mean ± SEM, control, n=15: 15.31x10^-2^ ± 2.54 x10^-2^, non-ICU, n=4: 8.33 x10^-2^ ± 0.68 x10^-2^, ICU-S, n=17: 7.28 x10^-2^±,0.74 x10^-2^ ICU-NS, n=29: 7.32 x10^-2^ ± 0.66 x10^-2^) or upon Complex-I (**Figure 3C**, ICU-S vs. control, p=8.7x10^-5^; ICU-NS vs. control, p=1.7x10^-5^, where Mean ± SEM, control, n=15: 18.98x10^-2^ ± 3.37x10^-2^, non-ICU, n=4: 9.28x10^-2^ ± 1.25x10^-2^, ICU-S, n=17: 7.19x10^-2^ ± 0.8x10^-2^, ICU-NS, n=29: 7.37x10^-2^ ± 0.76x10^-2^) or complex-II stimulations (**Figure 3D**, ICU-S vs. control, p=8x10^-4^; ICU-NS vs. control, p=2x10^-4^, where Mean ± SEM, control, n=15: 18.53x10^-2^ ± 3.38x10^-2^, non-ICU, n=4: 10.44x10^-2^ ± 1.9x10^-2^, ICU-S, n=17: 7.39x10^-2^ ± 0.8x10^-2^, ICU-NS, n=29: 7.57x10^-2^ ± 0.77x10^-2^). However, complex-IV activity was independent of the disease state (not shown) hinting that the observed respiratory depression is not due to levels of expression of mitochondrial ETC complexes. This was further demonstrated as shown in **supplementary Figure S1** through the comparisons of gene and protein expression profiles of mitochondrial ETC complexes in representative subsets (n=3-9) from all groups. We also observed a significant decrease in citrate synthase activity, a marker often used to reflect mitochondrial density, in ICU-hospitalized patients (**Figure 3E**, ICU-S vs. control, p=4x10^-4^; ICU-NS vs. control, p=0.0034, where Mean ± SEM, control, n=15: 2.37x10^-2^ ± 0.5x10^-2^, non-ICU, n=2: 1.26x10^-2^ ± 0.13x10^-2^, ICU-S, n=15: 0.29x10^-2^ ± 0.12x10^-2^, ICU-NS, n=21: 0.75x10^-2^ ± 0.27x10^-2^).

To explore if COVID-19-induced decline in mitochondria performance is related to qualitative mitochondrial morphological changes we analyzed TEM images of representative control (n=2, 96 mitochondria), ICU-S (n=1, 48 mitochondria), ICU-NS (n=3, 144 mitochondria); **Figures 3F–H**. Mitochondria from non-survivors exhibited significantly increased average cross-sectional area (**Figure 3G**, ICU-NS vs. control, p=0, where Mean ± SEM, Control: 157.81 ± 7.28, ICU-S: 157.22 ± 5.91, ICU-NS: 221.67 ± 6.73) and circularity (**Figure 3H**, ICU-NS vs. control, p=0.022, where Mean ± SEM, Control: 6.48x10^-2^ ± 0.43x10^-2^, ICU-S: 5.7x10^-2^ ± 0.23x10^-2^, ICU-NS: 7.77x10^-2^ ± 0.31x10^-2^) relative to controls’ and survivors’ mitochondria. These results suggest that non-survivors’ mitochondria are functionally and morphologically stressed with swollen and more-circular phenotypes.

### Severity-associated platelet phenotype showing hyperpolarized mitochondria, increased ROS, and remarkably reduced cytosolic calcium

**Figure 4:**
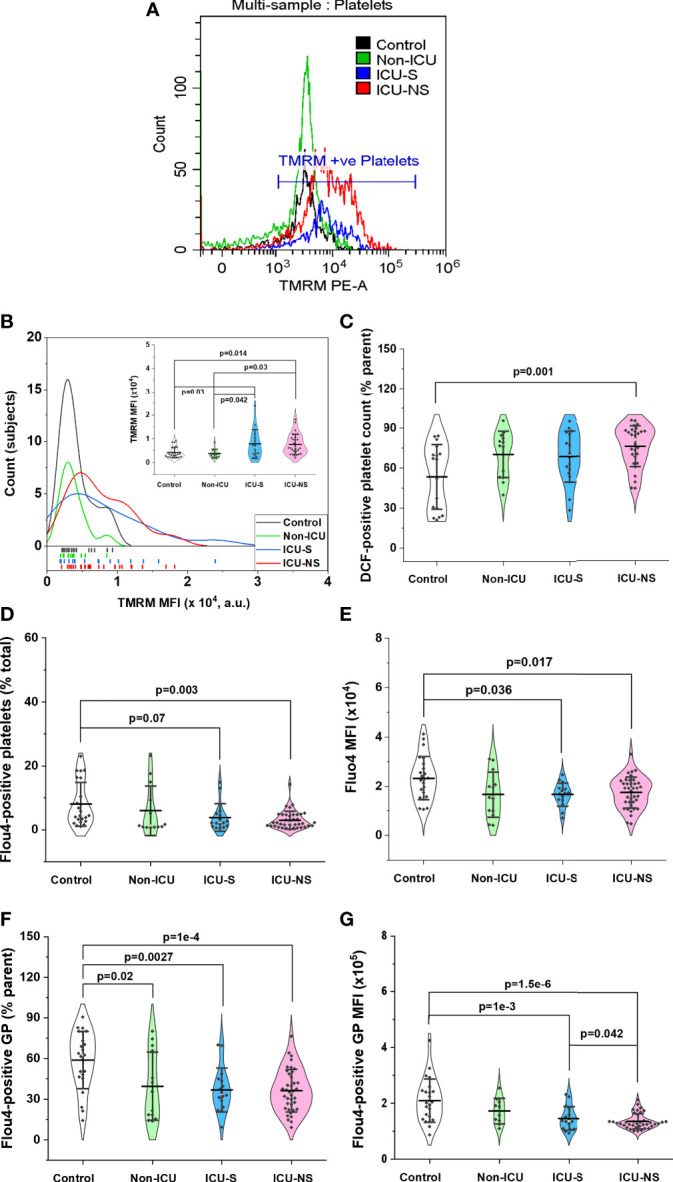
Changes in platelets’ mitochondrial transmembrane potential, ROS levels, and intracellular calcium in relation to severity and mortality outcomes. **(A)** Representative flow cytometric histograms comparing TMRM fluorescence for all groups. **(B)** When the distribution of TMRM mean fluorescence intensity were compared for all groups, both ICU-S and ICU-NS showed hyperpolarized mitochondria (n=23, 12, 16, and 29 for control, non-ICU, ICU-S, ICU-NS; respectively). DCF staining revealed increased levels of ROS in platelets (% parent) **(C)** of ICU-NS group (n=17, 13, 16, and 28 for control, non-ICU, ICU-S, ICU-NS; respectively). Quantification diagrams revealed significantly decreased counts of calcium-positive platelets **(D)**, n=23, 13, 16, and 38 for control, non-ICU, ICU-S, ICU-NS; respectively) and intracellular Ca^2+^ levels **(E)**, n=23, 13, 15, and 37 for control, non-ICU, ICU-S, ICU-NS; respectively) in platelets from ICU -hospitalized groups relative to control group. When the counts of calcium-positive giant platelets **(F)** and intracellular Ca^2+^ levels in giant platelets **(G)** were compared for all groups, ICU -hospitalized groups showed statistically significant increase in calcium-positive cell count and mean fluorescence intensity relative to control group (n=23, 13, 16, and 38 for control, non-ICU, ICU-S, ICU-NS; respectively). Multiple comparisons were carried out using ANOVA followed by Tukey test and p values are given. Data plotted as mean ± SD.

Previous studies suggested that mitochondrial hyperpolarization represents a key event in platelet activation and remodeling (21) in addition to increased formation of cytosolic reactive oxygen species (37). Furthermore, numerous studies highlighted a pivotal role for intracellular calcium in exocytosis through the direct activation of synaptic proteins with defined calcium-binding domains and/or the activation of protein kinases controlling granule secretion in activated platelets [e.g (38, 39).]. However, these molecular parameters were not adequately addressed in the context of SARS-CoV-2 viral infection. We used flow cytometry to explore if the observed mitochondrial dysfunction in critically-ill COVID-19 patients affects platelet function through modulating intracellular levels of ROS and calcium. First, CD42b-positive platelet populations that were simultaneously positive for TMRM staining were quantified to qualitatively reflect ΔΨ_m_, while intracellular ROS were assessed through DCF-positive cells, and intracellular [Ca^2+^] through Fluo4-positive staining as detailed in the methods’ section. Distribution of TMRM mean fluorescence intensity indicates hyperpolarized mitochondria in both ICU-S and ICU-NS in **Figures 4A, B**, ICU-S vs. control, p= 0.03; ICU-NS vs. control, p=0.0145. (Mean ± SEM, control, n=23: 0.42x10^4^ ± 0.05x10^4^, non-ICU, n= 12: 0.37x10^4^ ± 0.05x10^4^, ICU-S, n=16: 0.78x10^4^ ± 0.15x10^4^, ICU-NS, n=29: 0.76x10^4^ ± 0.08x10^4^). This was associated with increased ROS-positive platelets (% parent, **Figure 4C**, ICU-NS vs. control, p=0.001; Mean ± SEM, control, n=17: 53.48 ± 5.89, non-ICU, n= 13: 70.25 ± 4.82, ICU-S, n=16: 68.58 ± 4.80, ICU-NS, n=28: 76.22 ± 2.90). However, both counts of calcium-positive cells and levels of intracellular Ca^2+^ were significantly reduced in critically-ill patients’ platelets (**Figure 4D**, ICU-NS vs. control, p=0.003; Mean ± SEM, control, n=23: 8.0 ± 1.44, non-ICU, n=13: 5.98 ± 2.16, ICU-S, n=16: 3.81 ± 1.07, ICU-NS, n=38: 2.99 ± 0.45) (**Figure 4E**, ICU-S vs. control, p=0.036; ICU-NS vs. control, p=0.017; Mean ± SEM, control, n=23: 2.33x10^4^ ± 0.18x10^4^, non-ICU, n= 13: 1.66x10^4^ ± 0.26x10^4^, ICU-S, n=15: 1.66x10^4^ ± 0.12x10^4^, ICU-NS, n=37: 1.74x10^4^ ± 0.10x10^4^). We also analyzed giant platelet populations for the same parameters including transmembrane potential, ROS, and intracellular calcium. This analysis confirmed that giant platelets preserve profiles of these parameters similar to the general platelet populations. In **Figures 4F, G** we show that COVID-19 significantly reduces the number of Ca^2+^-positive giant platelets as well as diminished intracellular calcium levels especially in critically ill patients (**Figure 4F**, non-ICU vs. control, p=0.02; ICU-S vs. control, p=0.003; ICU-NS vs. control, p=1x10^-4^; Mean ± SEM, control, n=23: 58.90 ± 4.39, non-ICU, n=13: 39.59 ± 6.98, ICU-S, n=16: 36.88 ± 4.04, ICU-NS, n=38: 36.33 ± 2.54) (**Figure 4G**, ICU-S vs. control, p=0.001; ICU-NS vs. control, p=1.8x10^-6^; ICU-NS vs. non-ICU, p=0.042; Mean ± SEM, control, n=23: 2.10x10^5^ ± 0.16x10^5^, non-ICU, n=13: 1.80x10^5^ ± 0.14x10^5^, ICU-S, n=16: 1.46x10^5^ ± 0.11x10^5^, ICU-NS, n=38: 1.36x10^5^ ± 0.04x10^5^).

### Non-survivors exhibit hyper activated platelets that show a sign of impaired exocytosis

**Figure 5:**
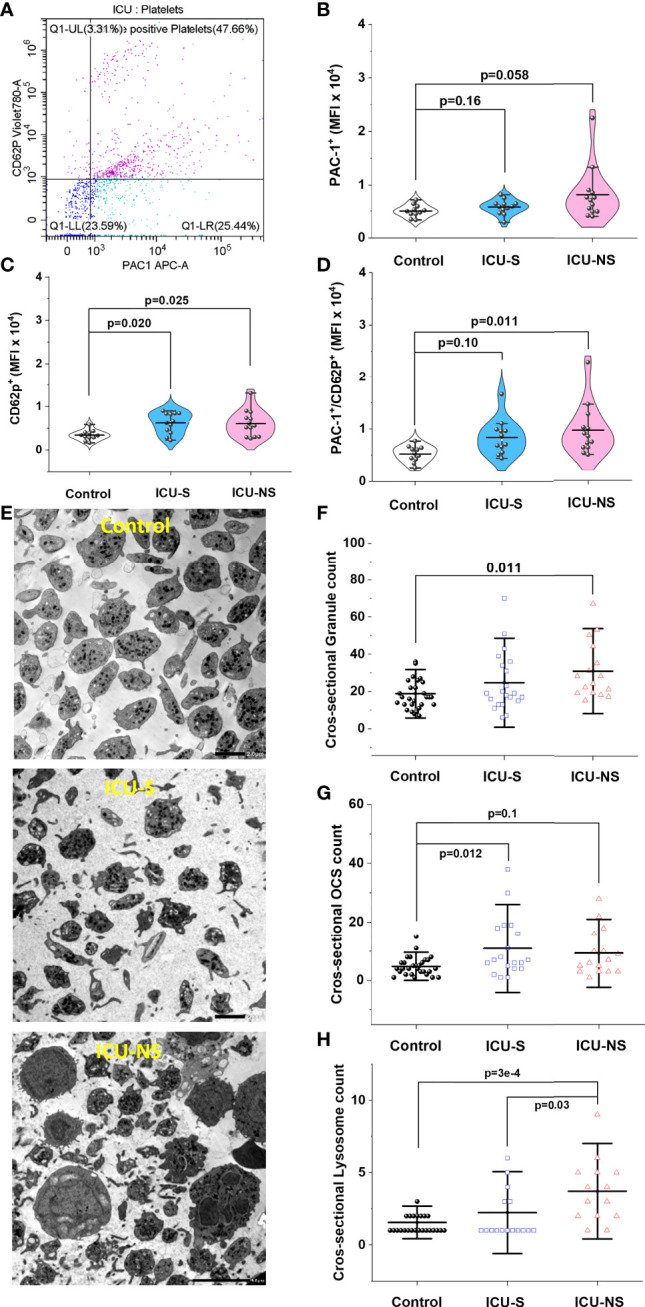
Increased platelet activation in severe COVID-19 patients. **(A)** Representative flow cytometric scatter diagram of PAC1 and CD62P double positive populations of platelets for severe patient. **(B)** A violin plot showing a trend of increase in PAC1 expression in ICU-hospitalized patients relative to control group (n=11, 12, and 13 for control, ICU-S, ICU-NS; respectively). **(C)** A violin plot showing significant increase in CD62P mean fluorescence intensity in both ICU-S and ICU-NS groups when compared to control group (n=11, 12, and 13 for control, ICU-S, ICU-NS; respectively). **(D)** When the MFI of CD62P/PAC1 were compared for all groups, only platelets of ICU-NS showed significantly higher MFI of double positive populations when compared to control group (n=11, 12, and 13 for control, ICU-S, ICU-NS; respectively). **(E)** Representative TEM images for control, ICU-S and ICU-NS groups showing a shift from a resting discoid shape in control subjects to an activated state with numerous pseudopodia in severe COVID-19 patients. **(F)** A diagram showing a significant increase in total granule counts (α- + dense-granules) in ICU-NS group compared to control group (n=3-4 subjects from each group and analyzed platelets = 31, 21, 16 for control, ICU-S, and ICU-NS; respectively). **(G)** A diagram showing a significant increase in open canalicular structures (OCS) in the ICU-S group relative to control group (n=3-4 subjects from each group and analyzed platelets = 29, 19, 16 for control, ICU-S, and ICU-NS; respectively). **(H)** A diagram showing significantly increased count of lysosomes per platelet of the ICU-NS group relative to control and ICU-S groups (n=3-4 of each group and analyzed platelets = 27, 17, 14 for control, ICU-S, and ICU-NS; respectively). Multiple comparisons were carried out using ANOVA followed by Tukey test and p values are given. Data plotted as mean ± SD.

Our results so far highlight cellular characteristics suggestive of increased platelets’ activation in COVID-19 patients. To confirm this, we employed flow cytometry technique to analyze platelet activation in their natural environment; i.e. in whole blood immediately after collection. We explored the dependence of the level of expression of platelet activation marker CD62P (P-selectin) and the activation of integrin receptor GP IIb/IIIa (PAC1) on disease severity and mortality outcome (**Figures 5A–D**). Although both markers are frequently used in the literature to assess platelet activation, CD62P was reported to be a more reliable marker compared with PAC1 for measuring platelet activation (40). Indeed, while platelets of both ICU-S and ICU-NS groups showed a trend of increase in PAC1 expression (**Figure 5B**; Mean ± SEM, control, n=11: 0.50x10^4^ ± 0.03x10^4^, ICU-S, n=12: 0.57x10^4^ ± 0.04x10^4^, ICU-NS, n=13: 0.81x10^4^ ± 0.14x10^4^), they significantly exhibited an increased CD62P mean fluorescence intensity when compared with control subjects (**Figure 5C**, ICU-S vs. control, p= 0.020; ICU-NS vs. control, p=0.025; Mean ± SEM, control, n=11: 0.34x10^4^ ± 0.04x10^4^, ICU-S, n=12: 0.62x10^4^ ± 0.07x10^4^, ICU-NS, n=13: 0.61x10^4^ ± 0.08x10^4^). Only platelets of non-survivors showed significant increase in MFI of double positive populations (**Figure 5D**, ICU-NS vs. control, p=0.011; Mean ± SEM, control, n=11: 0.52x10^4^ ± 0.05x10^4^, ICU-S, n=12: 0.84x10^4^ ± 0.1x10^4^, ICU-NS, n=13: 0.98x10^4^ ± 0.13x10^4^). In tune with this observation, TEM images in **Figures 1G** and **5E** demonstrate that platelets exhibit a shift from a resting discoid shape prevailing in control to an activated state with numerous pseudopodia in critically ill patients. Inspection of the same images hinted at increased levels of granules that are packed within platelets of COVID-19 patients, which, along with reduced intracellular calcium, are hallmarks of affected exocytosis (41, 42).

We therefore identified and counted α-granules, dense-granules, open canalicular structures (OCS), and lysosomes in magnified TEM images of randomly selected platelets from all groups (n= 3-4 from each group; n=31, 21, and 16 platelets for control, ICU-S, and ICU-NS; respectively); **Figures 5F–H**. To avoid ambiguity regarding granule assignments we analyzed total granule counts (α- + dense-granules) in TEM-acquired platelets cross-sectional images which showed significant increase in ICU-NS group relative to control group (**Figure 5F**, ICU-NS vs. control, p=0.011; Mean ± SEM, control, n=31: 18.84 ± 1.56, ICU-S, n=21: 24.67 ± 3.48, ICU-NS, n=16: 30.69 ± 3.81). For the same analyzed platelets, we observed an increased trend in counts of OCS/platelet in critically-ill patients that reached significance only in the ICU-S group (**Figure 5G**, ICU-S vs. control, p=0.012; Mean ± SEM, control, n=29: 4.79 ± 0.59, ICU-S, n=19: 10.95 ± 2.31, ICU-NS, n=16: 9.31 ± 1.95). Finally, platelets of the ICU-NS group showed significantly increased count of lysosomes per platelet relative to control and ICU-S groups (**Figure 5H**, ICU-NS vs. control, p=3x10^-4^; ICU-S vs. ICU-NS, p=0.03; Mean ± SEM, control, n=27: 1.56 ± 0.145, ICU-S, n=17: 2.24 ± 0.46, ICU-NS, n=14: 3.71 ± 0.59). These results are taken to suggest an impaired exocytosis in critically-ill COVID-19 patients especially those that didn’t survive.

### Platelet activation in severe COVID-19 patients is associated with increased tendency to aggregate with neutrophils but not with monocytes or T-lymphocytes

**Figure 6:**
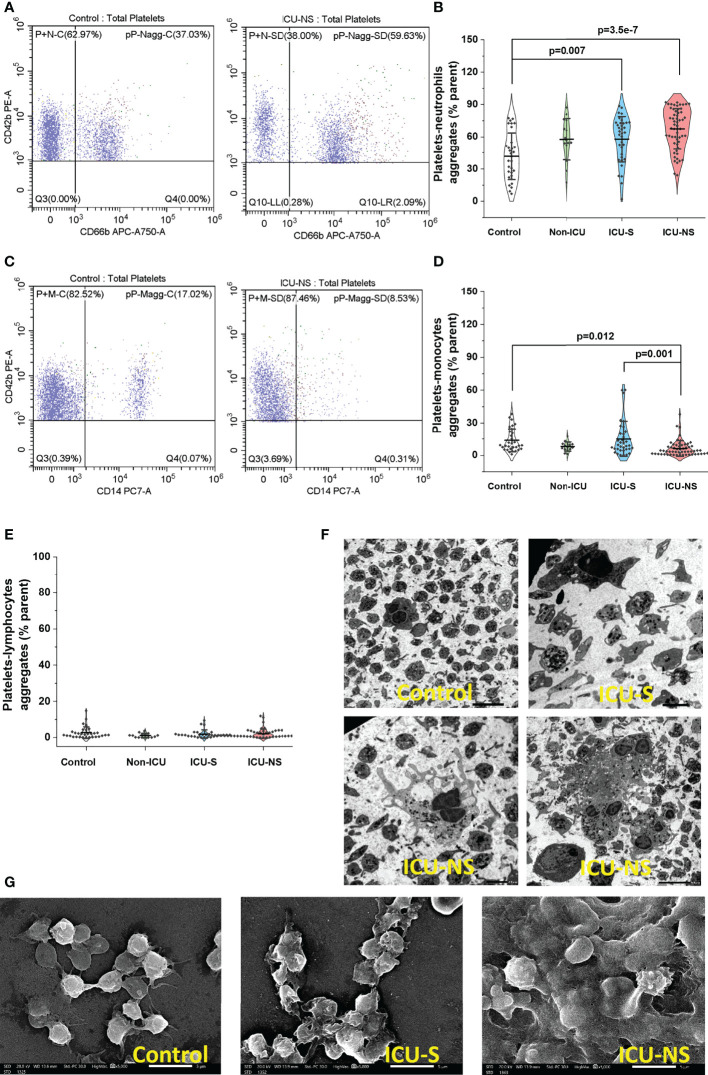
Platelet activation in severe COVID-19 patients is associated with increased tendency to aggregate with neutrophils. **(A)** Representative flow cytometric scatter plots of CD42b/CD66b double positive populations in control and ICU-NSgroups. **(B)** A violin plot showing significant increase in the percentage of CD42b/CD66b double positive populations in ICU patients (n=31, 15, 35, and 57 for control, non-ICU, ICU-S, ICU-NS; respectively). **(C)** Representative flow cytometric diagrams of CD42b/CD14 double positive populations in control and ICU (NS) groups. **(D)** A violin plot showing significant decrease in the percentage of CD42b/CD14 double positive populations in ICU-NS relative to controls, and relative to ICU-S group (n=31, 15, 35, and 57 for control, non-ICU, ICU-S, ICU-NS; respectively). **(E)** When the platelet-lymphocyte aggregation (CD42b/CD3 double positive populations) were compared for all groups, no statistically significant differences were observed between control, non-ICU, ICU-S, and ICU-NS (n=31, 15, 28, and 54 for control, non-ICU, ICU-S, ICU-NS; respectively). **(F)** Representative TEM **(F)** and SEM **(G)** images showing enhanced homo- and heterotypic aggregate formation in the ICU-NS group. Multiple comparisons were carried out using ANOVA followed by Tukey test and p values are given. Data plotted as mean ± SD.

Activated platelets may modulate inflammation *via* their receptor-dependent interactions with leukocytes and the subsequent exocytosis of bioactive mediators such as inflammatory cytokines. We asked if the above-described COVID-19-associated changes in molecular, mitochondrial, morphological, and activation profile exacerbate heterotypic platelet-leukocyte interactions especially in critically-ill patients. As previously reported, neutrophils-lymphocyte-ratio (NLR) and platelets-lymphocytes-ratio (PLR) showed systemic increase with severity and mortality (43); **supplementary Figure S2**. Additionally, in spite of administering anticoagulants in nearly half of the studied hospitalized patients (**Table 2**), flow cytometry analyses of whole blood samples freshly collected from ICU patients showed remarkable increase in the percentage of CD42b/CD66b double positive populations (platelet-neutrophil aggregates; **Figures 6A, B**, ICU-S vs. control, p=0.008; ICU-NS vs. control, p=3.5x10^-7^; Mean ± SEM, control, n=31: 41.77 ± 3.92, non-ICU, n=15: 57.75 ± 4.97, ICU-S, n=35: 57.89 ± 3.54, ICU-NS, n=57: 67.48 ± 2.48). Ironically, heterotypic platelet-monocyte aggregation (CD42b/CD14 double positive populations) was actually significantly lower in non-survivors’ case not only relative to controls, but also to survivors’ group (**Figures 6C, D** **)**; ICU-NS vs. control, p=0.012; ICU-NS vs. ICU-S, p=0.001; Mean ± SEM, control, n=31: 13.86 ± 1.90, non-ICU, n=15: 8.14 ± 1.11, ICU-S, n=35: 15.23 ± 2.72, ICU-NS, n=57: 6.43 ± 0.95). No tendency for altered platelet-lymphocyte aggregation (CD42b/CD3 double positive populations) was observed between any of the studied groups, **Figure 6E**. Indeed, transmission (**Figure 6F**) and scanning (**Figure 6G**) electron microscopy images of fixed buffy coats show the increased tendency of homo- and heterotypic aggregate formation especially in the ICU-NS group.

## Discussion

Over the track of evolutionary transition of invertebrates (ectotherms) to vertebrates (endotherms), platelets acquired new functions probably to cope with reduced oxygen levels during the Permian/Triassic period which eventually selected for endothermic animals (44). This appears to have increased aerobic capacity, vascularization, systemic blood pressure, and shear forces leading to anucleation of platelets (44). Metabolism and mitochondria are thus critical factors that have associated and perhaps directed platelets’ acquisition of novel functions (45). Instead of directly killing pathogens, platelets assumed the role of immune modulators, mediating inflammatory responses to pathogens, and regulation of hemostasis and blood coagulation. The roles of platelets in COVID-19-associated coagulopathy have been highlighted (2, 8, 9) but their modulatory role of the immune response following SARS-CoV-2 infection remains largely unattained. We designed the present study to explore the impact of COVID-19 severity and mortality on molecular factors that determine hemostatic and immune modulatory functions of platelets. We paid specific attention to knowledge gaps pertaining to metabolic remodeling and alterations of mitochondrial function inflected by disease severity and known to contribute to platelet-associated hemostatic and immune dysfunctions.

First, we assessed and matched morphologic as well as metabolic profiles in platelets of a relatively large prospective cohort of subjects including 31 controls, 15 non-ICU, and 95 ICU-hospitalized patients of whom 60 have died. Comorbidities such as cardiovascular disease and secondary infections have been linked to increased disease severity and death in patients with COVID-19 (46–48). Although the prevalence of comorbidities in our case was generally higher in the order ICU-NS>ICU-S>Non-ICU, the studied cohort didn’t show statistically significant association between comorbid conditions and COVID-19 severity. CRP levels may predict the severity of COVID-19 disease (49) however, this has not been the case in another study (50) like the present study where there was no significant difference between the CRP levels after transferring the patients to ICU. Creatinine levels appear to be significantly associated with increased disease severity which confirms the results of prior studies that demonstrated the use of creatinine as an indicator of prognosis in patients with COVID-19 (51, 52). In this study both ICU-S and ICU-NS patients have greater leukocytes count than Non-ICU group. A previous study reported that critically ill individuals with COVID-19 have greater leukocyte numbers than healthy people (53, 54). Laboratory data obtained from the collection site (**Table 3**) showed that the number of platelets of ICU patients was generally lower than the other groups which is in harmony with another meta-analysis showing reduced platelet counts in very ill individuals (55). In tune with this, the total number of platelets determined in our lab using flow cytometry showed similar trends (**Figure 1B**).

Multiple studies have shown that increased mean platelet volume (MPV) may reflect thrombotic risk (56, 57), and that MPV can serve as a marker of platelet activity in patients with pulmonary embolism (58). In a very recent large-scale clinical assessment study, cardiovascular events were found to increase early after COVID-19 mainly from pulmonary embolism, atrial arrhythmias, and venous thrombosis (3). Our morphometric analyses using flow cytometry and electron microscopy showed that critically-ill patients possess platelets with increased MPV, inner complexity, and increased proportions of giant platelets thus substantiating the role of platelets in COVID-19 cardiovascular complications.

Despite the pivotal role played by mitochondria and metabolism in determining platelet function and immune modulation (15, 16), very scarce efforts have been devoted to investigate these factors in the context of COVID-19 pathology. To the best of our search ability, only one study assessed mitochondrial respiratory function and showed that platelet mitochondrial function and endogenous coenzyme Q10 levels are reduced post COVID-19 infection (59). The dynamic functions of platelets require complementary metabolic machineries to meet energetic demand during normal physiology which interchangeably involve both glycolysis and mitochondrial oxidative phosphorylation. Our analysis thus included metabolic pathway profiling and detailed mitochondrial respiratory activities in all groups. We showed here that an overall metabolic depression and impaired metabolic flexibility are hallmarks of platelets freshly isolated from COVID-19 patients with increasing severity. Based on reported platelets’ metabolic flexibility, and contrary to our expectation, glycolytic activities in critically-ill patients’ platelets were also remarkably depressed. This can be suggested to hamper the ability of platelets to switch freely between bioenergetic pathways in response to functional demands and dynamic changes in shear stress and coagulations. In spite of a variable and weak tendency towards reduced levels of mitochondrial ETC complexes’ gene and protein expressions in COVID-19 patients (**Supplementary Figure S1**) mitochondrial respiratory function was found to be remarkably depressed in critically-ill patients. Furthermore, electron microscopy analyses revealed increased average area and increased circularity of mitochondria in platelets of those patients. These results suggest stressed and functionally impaired mitochondria especially in critically ill patients.

Mitochondria are critical organelles that are established to play a multifaceted role surpassing the conventional powerhouse and includes cell signaling, redox modulation, activation, and death pathways in platelet physiology and pathophysiology. For example, platelet mitochondria are pivotal to the activation and interaction with leukocytes through regulation of calcium and phosphatidylserine externalization (19, 20, 18). Studies indicated that the transition of platelets to pro-coagulant phenotype is mediated by hyperpolarized mitochondria (21), extracellular calcium entry (22), and mitochondrial permeability transition pore (mPTP) opening (22) and this is associated with intra-platelet ROS elevation (23). Here we found that critically-ill platelets exhibit hyperpolarized mitochondria and consistently increased number of platelets expressing intracellular ROS. Generally, inhibition of oxidative phosphorylation leads to hyperpolarized mitochondria and also increases ROS production presumably to enhance bactericidal activities in immune cells (24, 60). Concomitantly, intracellular calcium levels were significantly lower in platelet populations but particularly so in giant platelets of critically ill patients. It can be argued that reduced intracellular calcium contribute to impaired exocytosis. We suggest here that excessive uptake of calcium by hyperpolarized mitochondria in critically ill COVID-19 patients leads to abnormally depleted calcium levels in those patients’ platelets. Although this is partially supported by the observation of swollen mitochondria, the current results cannot rule out other mechanisms affecting calcium levels in platelets. For example, depressed metabolic activities in critically ill patients may impair the function of calcium channels requiring ATP for their activation. Impaired calcium dynamics leads to impaired nerve transmission and pathology of the peripheral nervous system, with mechanisms shared by platelets and causing bleeding tendency of zinc deficiency (39, 61). Consistently, impaired mobilization and significantly lowered levels of intracellular calcium in neonatal platelet has been reported (42) and was suggested more recently to cause impaired granule trafficking and secretion despite signs of platelets hyper activation (41).

The importance of platelets as parts of the immune system whether innate or adaptive is increasingly recognized (4–6). In fact, evidence showed that platelets’ activation profile during thrombin-regulated hemostatic response to pathogens distinctly differs from that following immune stimulation; e.g. by TLR7-activating virus (7). We provide evidence that platelets of non-survivors exhibit hyper activated phenotype including a significant increase of specific surface markers and morphologic shift from a resting discoid shape into an activated state with numerous pseudopodia. To distinguish whether platelets activation is part of hemostatic and/or immune responses we analyzed platelet-leukocyte aggregations. That is, while hemostatic response is expected to involve platelet-platelet interactions, immune-activation leads to smaller platelet groups involving frequent interaction with leukocytes (5). Platelet-leukocyte interactions, especially with neutrophils and monocytes, are thus substantiated as core immune modulators. Here we report diverse aggregation tendencies of platelets with various leukocytes. Platelet-T-lymphocytes aggregation was not affected by severity or mortality outcomes, but platelet-neutrophil aggregation increased remarkably and that with monocytes decreased significantly in non-survivors. Aggregation of platelets with neutrophils is consistently observed in the contexts of an array of human conditions and is viewed as one of the most sensitive markers of platelet activation (10). Importantly, upon activation, platelets bind to neutrophils and trigger the release of cytokines and stimulates the formation of neutrophil extracellular traps (11, 12) known to contribute to the pathology of COVID-19 (62). However, the role of platelet-monocyte aggregates is still debatable with paradoxical roles of increased inflammation in the pathogenesis of sepsis (13), while being suggested to play anti-inflammatory role in the context of ulcerative colitis (14). In our hands, the remarkable reduction in platelet-monocyte aggregation in non-survivors potentiates the anti-inflammatory character of these aggregates, which requires further studies to verify and understand the downstream effects of this observation.

In summary, in this study, we uniquely employed multiple complementary approaches and sensitive technologies to assess the roles of metabolism and mitochondria in platelets’ molecular and cellular functions in a relatively large cohort of Non-ICU as well as ICU-hospitalized critically-ill COVID-19 patients. Taken together, our data suggest that hyperactive platelets with impaired exocytosis may be integral parts in the pathophysiology dictating severity and mortality in COVID-19 patients. This may guide further studies targeting exocytosis for boosting immune-modulatory functions of platelets in the context of SARS-CoV-2 infection.

## Data availability statement

The original contributions presented in the study are included in the article/**Supplementary Material**. Further inquiries can be directed to the corresponding authors.

## Ethics statement

The studies involving human participants were reviewed and approved by Children’s Cancer Hospital’s Institutional Review Board. The patients/participants provided their written informed consent to participate in this study.

## Author contributions

BY and AE performed experiments, analyzed results, and assisted in the manuscript preparation. Subjects’ recruitment, patients’ consents, clinical follow-up and assessments were coordinated by RE-M. MA-A, HA-S, ME, AK, SG, and MZ assisted in experimental work. MH analyzed clinical data. SA and EA-R conceived and directed the project, designed experiments, and analyzed data. SA obtained funding and wrote the manuscript. All authors read and approved the submitted manuscript.

## Funding

The present work was funded by the Association of Friends of the National Cancer Institute and the Children’s Cancer Hospital Foundation.

## Conflict of interest

The authors declare that the research was conducted in the absence of any commercial or financial relationships that could be construed as a potential conflict of interest.

## Publisher’s note

All claims expressed in this article are solely those of the authors and do not necessarily represent those of their affiliated organizations, or those of the publisher, the editors and the reviewers. Any product that may be evaluated in this article, or claim that may be made by its manufacturer, is not guaranteed or endorsed by the publisher.
