# Supplementary material for: Platelets’ morphology, metabolic profile, exocytosis, and heterotypic aggregation with leukocytes in relation to severity and mortality of COVID-19-patients
Source: Front Immunol. 2022 Nov 21;13:1022401. doi: 10.3389/fimmu.2022.1022401 (PMC9720295; doi:10.3389/fimmu.2022.1022401)

Supplementary Material

Platelets’ morphology, metabolic profile, and heterotypic aggregation with leukocytes in relation to severity and mortality of COVID-19-patients

Basma A. Yasseen,^1, †^ Aya A. Elkhodiry,^1, †^ Riem M. El-Messiery,^2^ Hajar El-sayed,^1^ Malak W. Elbenhawi,^1^ Azza G. Kamel,^1^ Shaimaa A. Gad ,^3^ Mona Zidan,^1^ Marwa S. Hamza^4^, Mohamed Al-ansary^5^, Engy A. Abdel-Rahman,^1,6,*^ and Sameh S. Ali^1,*^

# Supplementary Figures and Tables

**Supplementary Figure 1.** (A) Representative Western blot showing protein expression of mitochondrial electron transport chain complexes (ETC) in platelets of control, non-ICU, ICU-S, and ICU-NS. (B) Densitometric analyses of ETC complexes proteins normalized by b-actin in all groups. Values are calculated as relative intensity/µg of loaded protein normalized by b-actin expression and are given as mean ± SD (n=3-9 of each group represented). (C) When ETC complexes gene expressions in platelets were compared between groups, control, non-ICU, ICU-S, and ICU-NS, no significant differences were detected (n=6 of each group). Multiple comparisons were carried out using ANOVA followed by Tukey test and p values are given.

##
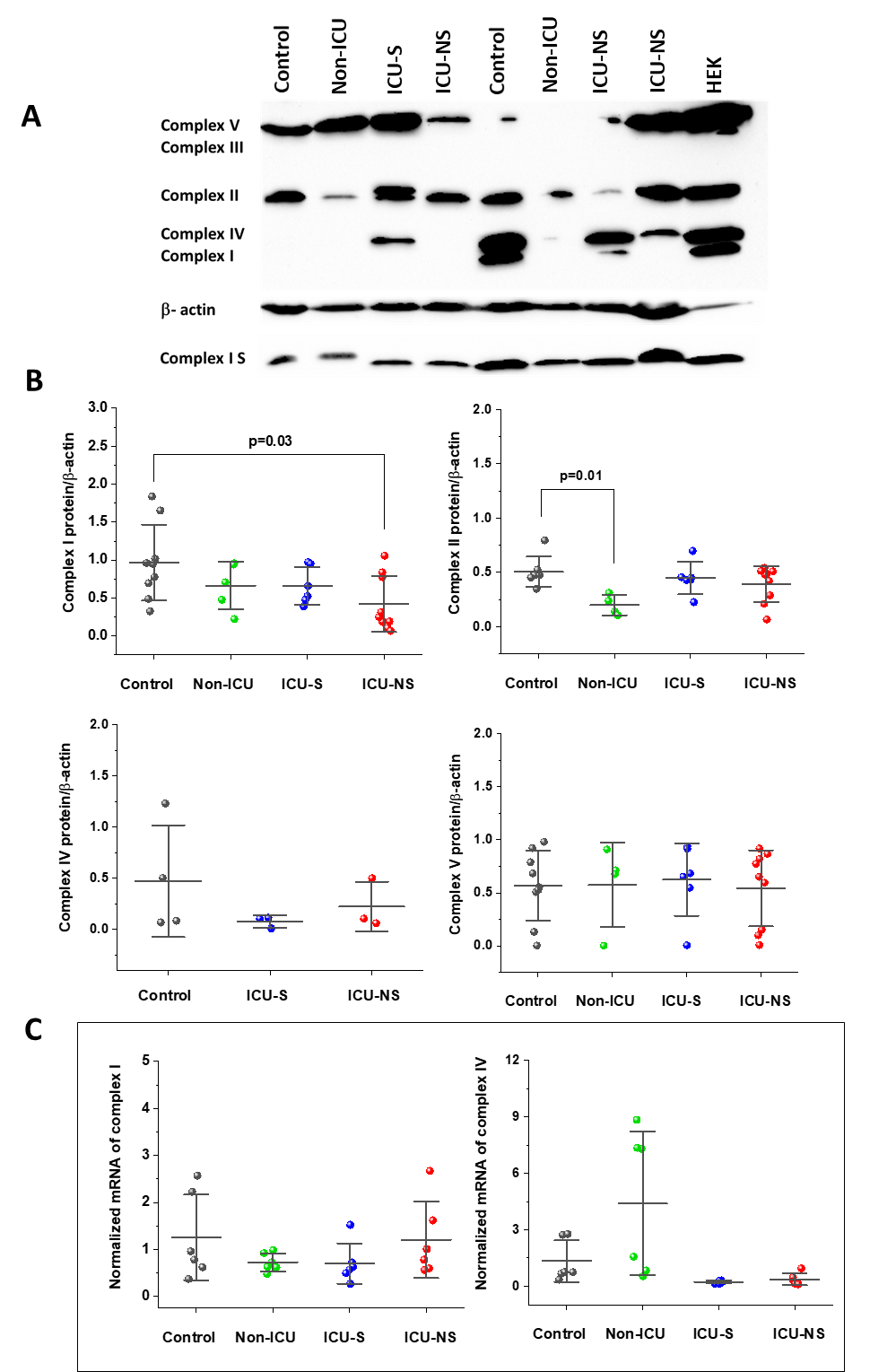


**Supplementary Figure 2.** Comparisons of immune cells subsets in all groups through neutrophil-lymphocyte ratio (A) and platelet-lymphocyte ratio.


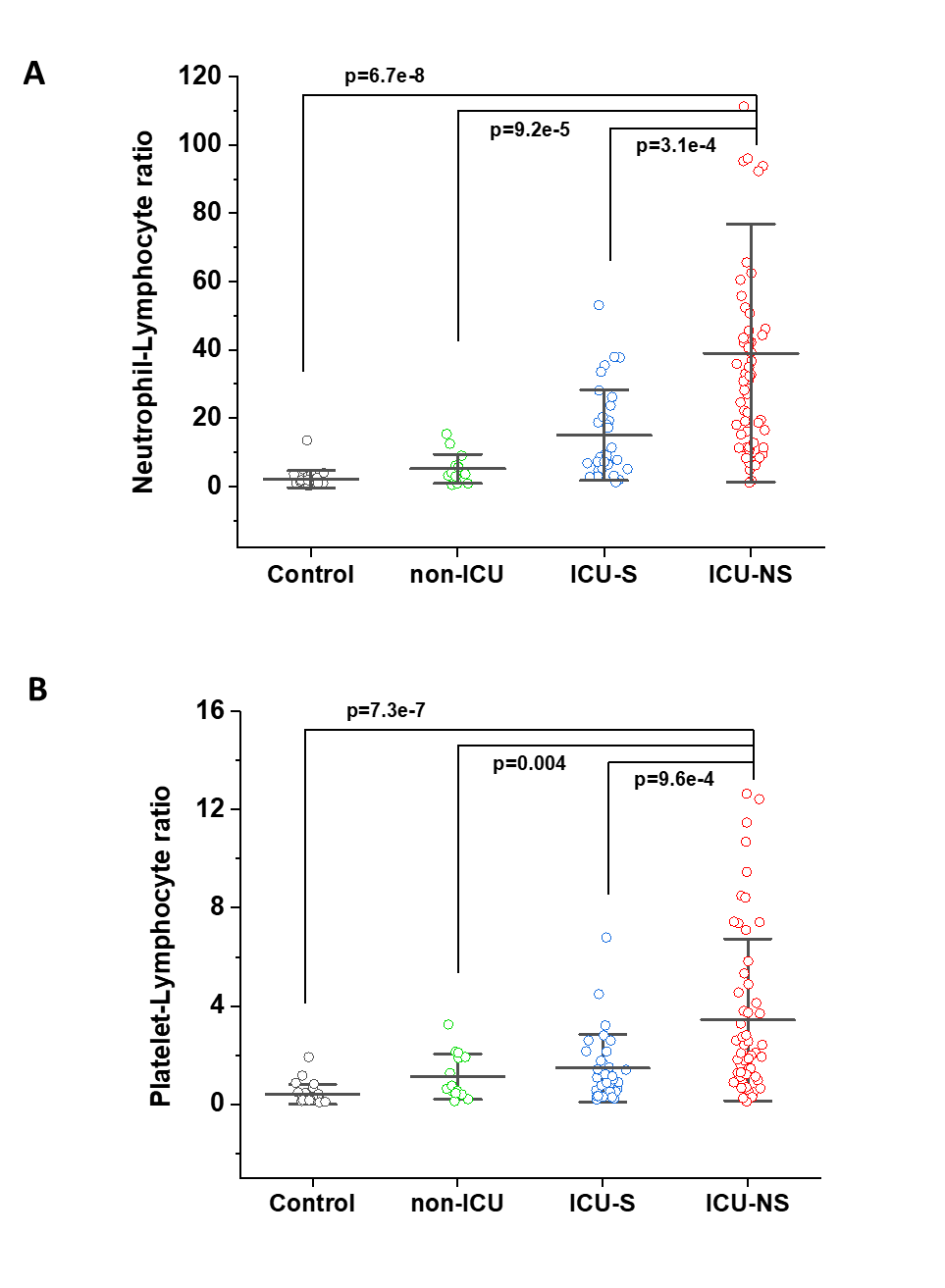


**Supplementary Figure 3**. TEM images for two control, two ICU-S, and two ICU-NS subjects showing additional examples of morphological changes associated with COVID-19 mortality. Dimensions of the scale bars are given on each image.


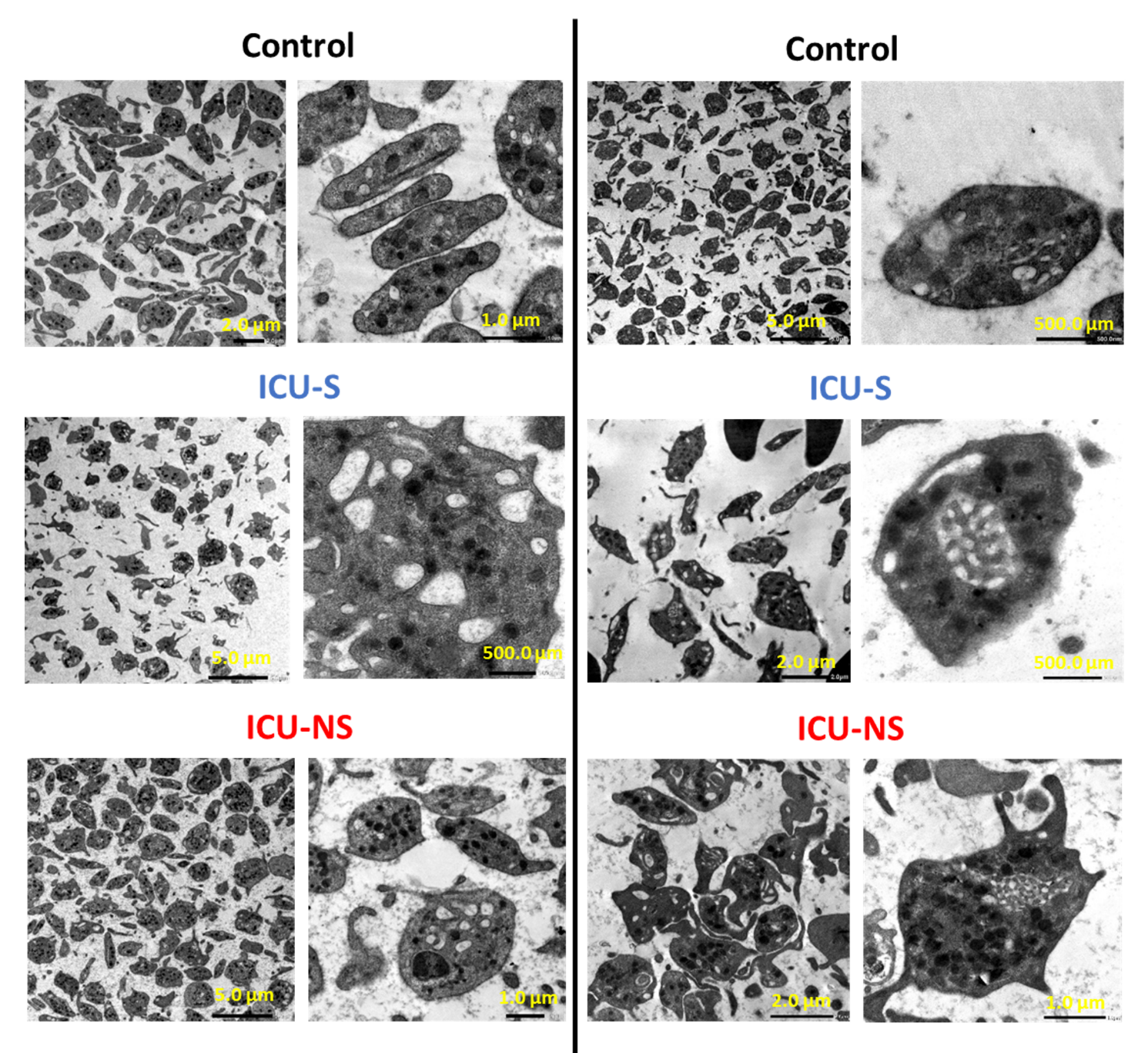

Supplement: Supplementary file 1 [file DataSheet_1.docx]
